# Hepatoid adenocarcinoma of the stomach – a different histology for not so different gastric adenocarcinoma: a case report

**DOI:** 10.1186/1477-7800-6-13

**Published:** 2009-08-12

**Authors:** Elisa Gálvez-Muñoz, Javier Gallego-Plazas, Verónica Gonzalez-Orozco, Francisco Menarguez-Pina, José A Ruiz-Maciá, Miguel A Morcillo

**Affiliations:** 1 Department of Medical Oncology, Hospital General Universitario de Elche, Elche, Alicante, Spain; 2 Department of General Surgery, Hospital Vega Baja, Orihuela, Alicante, Spain; 3 Department of Pathology, Hospital Vega Baja, Orihuela, Alicante, Spain

## Abstract

Hepatoid adenocarcinoma is an extrahepatic tumor characterized by morphological similarities to hepatocellular carcinoma. Hepatoid adenocarcinoma of the stomach is a cancer with an extremely poor prognosis with few cases reported. Here, we describe a 75-year-old Spanish man referred to our hospital with a history of abdominal pain, general fatigue, anorexia and sickness. Initial study revealed anemia, and computed tomography scan and abdominal ultrasonography showed multiple metastases to the liver with hepatocellular carcinoma characteristics in a liver with no cirrhotic change. Further study included a serum level of alpha-fetoprotein (AFP), which resulted markedly elevated, and a conclusive esophagogastroduodenoscopy describing an elevated tumour growing through the cardia and gastroesophageal junction with foci of necrosis and haemorrhage. Gastric biopsies of the tumor revealed poorly differenciated adenocarcinoma, with hepatoid differentiation. After a diagnosis of AFP-producing hepatoid adenocarcinoma of the stomach with multiple liver metastases was made, pallitive total gastrectomy, without liver resection, was performed. Patient recovered well after surgery, and entered into a palliative systemich chemotherapy protocol. Although this illness is recognized as having poor prognosis, the patient remains alive 8 months after the operation. Accurate diagnosis of hepatoid adenocarcinoma of the stomach is important, and should be suspected under certain circumstances. We describe this rare case of hepatoid adenocarcinoma of the stomach, and review the literature concerning the clinicopathological aspects.

## Background

Alpha-fetoprotein (AFP) level in serum, initially elevated due to fetal liver, yolk salk, and some fetal gastrointestinal cells production, rapidly decreases after birth [1]. AFP level is, however, elevated in patients with hepatocellular carcinoma and in those with other liver diseases associated with liver regeneration [2-4]. AFP-producing tumors, others than hepatocellular carcinoma, have been described [5-8]. First reported in 1970 by Bourreille *et al*. [10], AFP-producing gastric adenocarcinoma has now an incidence reported to be 1.3% – 15% of all gastric carcinomas [11,12]. In 1981, Kodama *et al*. described two histologic types of AFP-producing gastric carcinoma with medullary or papillotubular arrangements [13]. Later Ishikura *et al*. [14,15] proposed the term "hepatoid adenocarcinoma of the stomach" for primary gastric carcinomas that are characterized by both hepatoid differentiation and the production of large amounts of AFP. In 1986, the clinicopathologic entity was broadened to include gastric carcinoma showing hepatic differentiation without the production of AFP. Hepatoid adenocarcinomas have also been reported in several different organs, such as those occurring in the lung, pancreas and ovary, although gastric adenocarcinoma continuous to be one of the most commons of these tumours [5-9]. This type of tumour is a relatively rare gastric carcinoma, and is recognized as having poor prognosis, even if the tumour is diagnosed at an early stage. In this report, we describe a rare case of hepatoid adenocarcinoma of the stomach, with a review of the literature concerning clinicopathological aspects.

## Case presentation

A 75-year-old man presented at the emergency department of Vega Baja Hospital, Orihuela, Alicante, on February 2008, with of a 15-day history of abdominal pain, general fatigue, anorexia and sickness. His medical history included hypertension, adenomatous colonic polyposis, upper gastrointestinal bleeding secondary to agiodysplasia treated with enterectomy in 2005, obstructive pulmonary chronic disease related to hemp industry, and right adrenal adenoma since 2005.

**Figure 1:**
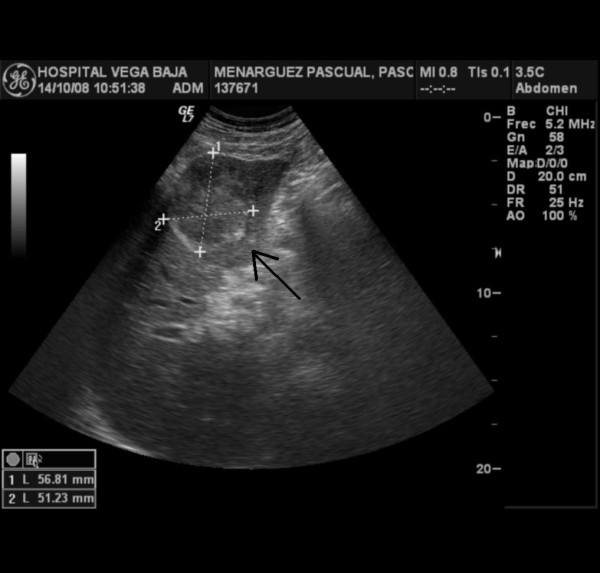
**Abdominal ultrasonoraphy showing liver with muliple nodules, biggest in left liver lobe (56 × 51 mm.), highly suspicious for hepatocellular carcinoma**.

**Figure 2:**
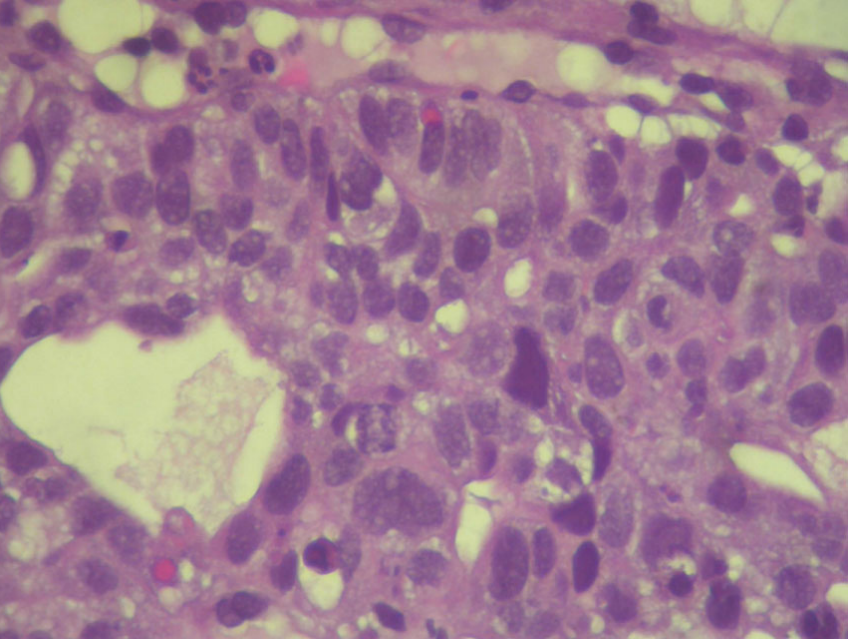
**Gastric biopsy 400×. Hemotoxylin Eosin staining**.

**Figure 3:**
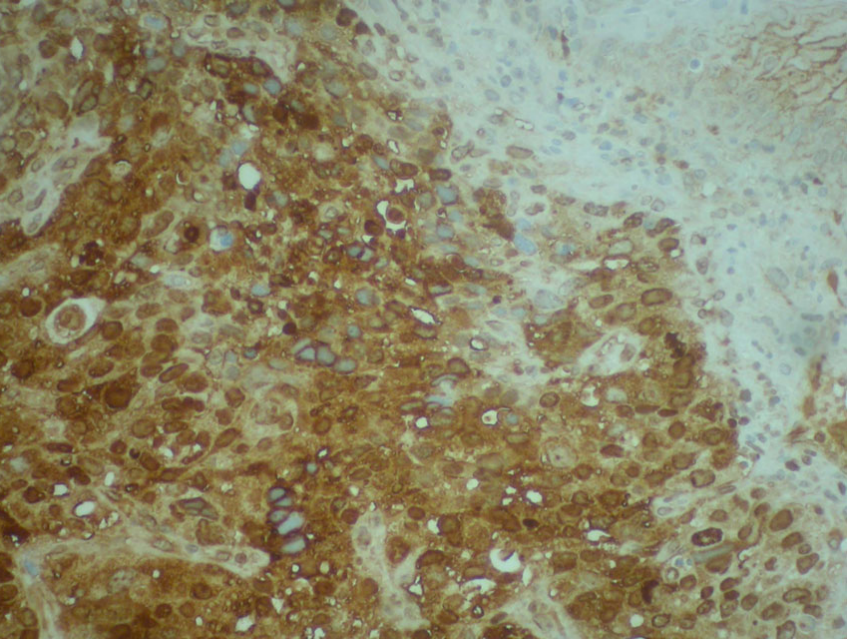
**Gastric biopsy with inmunohistochemical staining showing strongly positivity for AFP**.

On physical examination only paleness of skin and conjunctiva was detected. He was referred to Internal Medicine Department fur further study. Haematological investigations revealed anemia (Hemoglobine (Hb), 7.1 g/dl; hematocrit (Ht), 25% and high levels of AFP (4,500 UI/mL) and CEA (460 ng/mL). Chest and abdominal radiograph were negative. After blood transfussion, other image test were performed. Abdominal ultrasonoraphy showed liver with muliple nodules, biggest in left liver lobe (56 × 51 mm.), highly suspicious for hepatocellular carcinoma (Figure 1. Abdominal ultrasonoraphy showing liver with muliple nodules, biggest in left liver lobe,56 × 51 mm., highly suspicious for hepatocellular carcinoma). Esophagogastroduodenoscopy findings showed an elevated tumour growth through the cardia and gastroesophageal junction with foci of necrosis and haemorrhage. Gastric biopsy of the gastroesophageal junction revealed on hematoxylin eosin staining poorly differenciated adenocarcinoma, with probably hepatoid differentiation. The inmunohistochemical staining showed strongly positive for AFP (Figure 2. Gastric biopsy 400×. Hemotoxylin Eosin staining) (Figure 3. Gastric biopsy with inmunohistochemical staining showing strongly positivity for AFP). Further inmunohistochemical analysis revealed positive expression of CDX2 and CD10, as more than 10% of the carcinoma cells were stained.

**Figure 4:**
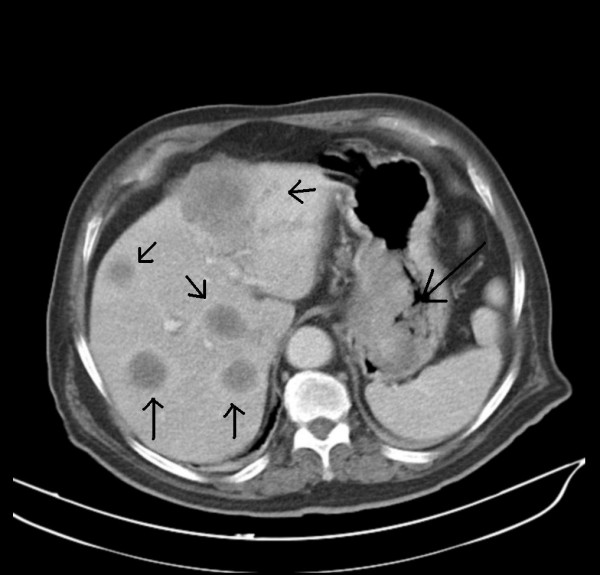
**Computed tomography showing multiple solid lesions suggestive of metastases in both lobes of the liver, and a thickening of the medial wall-less curvature of the stomach and lymph node swelling around the gastro-hepatic ligament suspicious of gastric neoplasia**.

With definite diagnosis of AFP-producing hepatoid gastric adenocarcinoma metastatic to the liver, patient was then referred to Clinical Oncology Department where, in order to complete pretreatment evaluation, a thoracic-abdominal-pelvic CAT was requested. Results of computed tomography revealed multiple solid lesions suggestive of metastases in both lobes of the liver. A thickening of the medial wall-less curvature of the stomach and lymph node swelling around the gastro-hepatic ligament suspicious of gastric neoplasia was also described (Figure 4. Computed tomography showing multiple solid lesions suggestive of metastases in both lobes of the liver, and a thickening of the medial wall-less curvature of the stomach and lymph node swelling around the gastro-hepatic ligament suspicious of gastric neoplasia).

**Figure 5:**
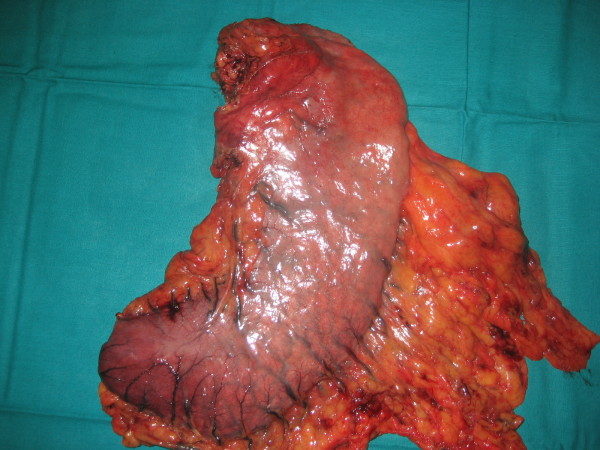
**Total gastrectomy specimen**.

**Figure 6:**
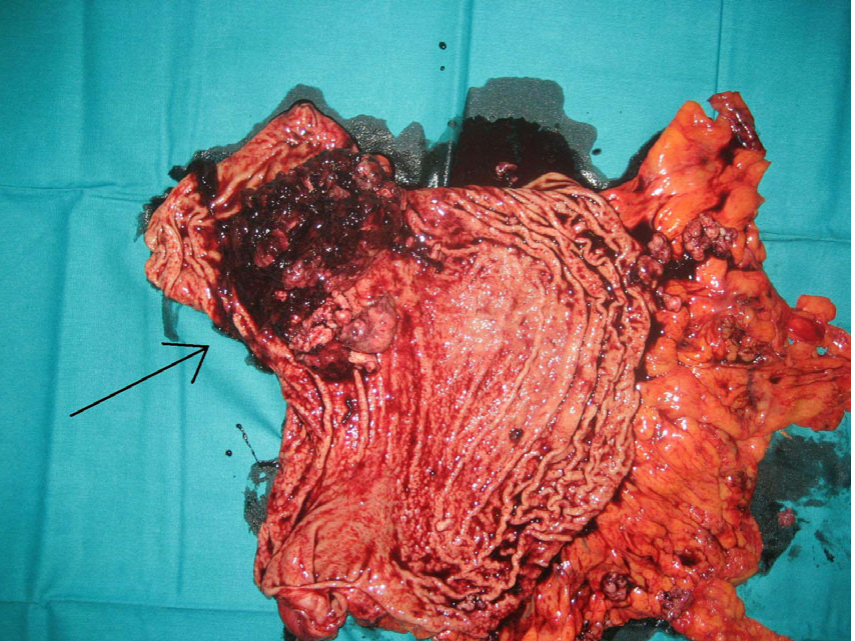
**Upper vision of opened gastrectomy specimen with tumor upper left**.

**Figure 7:**
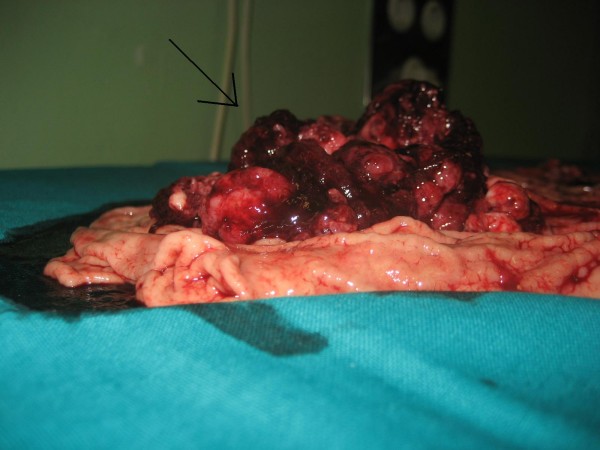
**Lateral vision of gastrectomy specimen showing excrecent tumor)**.

**Figure 8:**
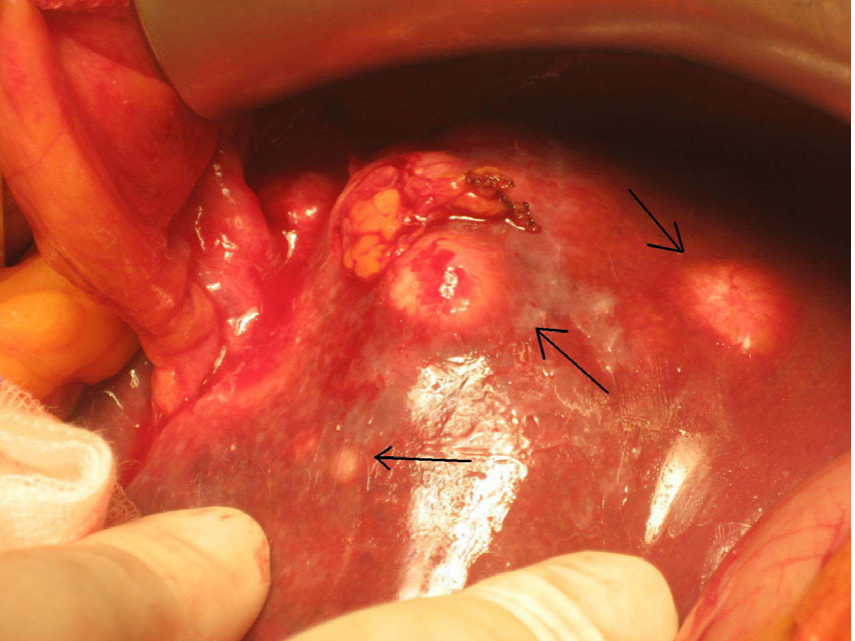
**Liver metastases were palpable and visible during surgery as they were in the liver surface)**.

The patient required meanwhile 6 blood transfusions because of persistent melena and referred symptomatic anemia so he was proposed first to palliative total gastrectomy. Surgery was succesfully completed with gastric resection including a bleeding polylobate endoluminal mass located in the cardias and gastroesophageal junction (Figure 5. Total gastrectomy specimen) (Figure 6. Upper vision of opened gastrectomy specimen with tumor upper left) (Figure 7. Lateral vision of gastrectomy specimen showing excrecent tumor). During surgery liver metastases were palpable and visible as they were in the liver surface (Figure 8. Liver metastases were palpable and visible during surgery as they were in the liver surface), but no surgical procedure besides palliative gastrectomy was performed. Anatomopathology analysis revealed a 9 cm big gastric hepatoid adenocarcinoma which penetrated serosa without invasion of adjacent structures (pT3), with no vascular invasion (V0). Regional lymph node assessment described 9 out of 16 lymph nodes with metastasis of hepatoid adenocarcinoma (pN2). Inmunohistoquemical analysis of the resected tumor was negative for CK7, CD 20, and cromogranine, while positive for CEA and AFP.

Patient soon recovered after surgery with no major complications besides requiring percutaneous drainage of subfrenic collection in the seventh day postoperative. Total Hospital stay for surgical procedure and management of subfrenic collection was of sixteen days. Patient was then referred again to Clinical Oncology Department. By that time patient complained for anorexia and gastrointestinal disorders supposedly related to recent surgery. He had lost eight kilograms since first admission into hospital 30 days before. As his karnofsky score was 70, with no major hematological or chemistry abnormalities, palliative chemotherapy consisting of cisplatin and capecitabine was advised. Patient since then has received six cycles of chemotherapy with stable disease on CAT and progressive reduction of AFP and CEA values on evaluation after third and sixth cycles. Tolerance to chemotherapy has been adequate with grade II hand-foot syndrome, grade II nausea, grade I diarrhea as most relevant toxicities, with no grade 3/4 toxicities reported. Further chemotherapy with the same cisplatin and capecitabine regimen is being administered.

## Discussion

Gastric hepatoid adenocarcinoma is a kind of tumour with unique histological characteristics. Although it has been reported in the lung, pancreas, uterus and ovary, it is most commonly seen in the stomach [5-9]. It is considered an AFP- producing malignant tumour. Kodoma *et al*. [12] described two histologic types of AFP-producing gastric carcinoma, based on inmunohistochemical staining; a medullary type, characterized by polygonal cells arranged in solid nest or sheets, with scattered large pleomorphic or multinucleated giant cells, and well differenciated papillary or tubular type with clear cytoplasm. The two types sometimes coexisted in a single tumour. The reported incidence of AFP-producing gastric carcinoma has been 1.3%–15% of all gastric carcinomas [11,12].

Why some gastric carcinomas show hepatoid differentiation and produce AFP has been the subject of some debate. Ishikura *et al*. [14,15] suggested that this may be due to the fact that the stomach and liver are both derived from primitive foregut of the embryo. Because of this, disturbances of differentiation ultimately may result in the development of foci of hepatocellular differentiation. Furthermore, there is sometimes confusion about whether hepatoid carcinoma originates from the stomach or the liver, because most patients show multiple liver metastasis preoperatively [16].

Hepatoid carcinoma contains a tubular adenocarcinoma that seems to develop "hepatoid features", but the relation between the tubular adenocarcinomatous and the hepatoid components remains unclear. A recent analysis from Kumashiro *et al*. [17] compared the cellular penotypes of 23 cases of hepatoid adenocarcinoma of the stomach having tubular adenocarcinomatous components with 69 cases of non-hepatoid adenocarcinoma of the stomach. Four phenotypic categories according to the inmunohistochemical results for CD10, MUC2, MUC5AC, and MUC6 were described for both components, tubular adenocarcinomatous and hepatoid, of hepatoid adenocarcinoma. The complete intestinal phenotype (CD10+, MUC5AC-, MUC6-), such is the case for our patient, was most frequently observed in the adenocarcinomatous and hepatoid components (61 and 65%, respectively). In contrast, no gastric phenotype (MUC5AC+, MUC6+, MUC2-, CD10-) was observed in any of the hepatoid adenocarcinoma components. The fact that positivity for CDX2 was predominant in all the adenocarcinomatous components, while negative in 39% of the hepatoid components, suggests that hepatoid adenocarcinoma arises from an adenocarcinoma with intestinal phenotype and that its hepatoid component is in some way related to reduced CDX2 expressión.

Case reports of "hepatoid adenocarcinoma of the stomach" have been previously reported in the literature. In 2001, Inawaga *et al*. [16] revised all these cases and published a report with the main clinical characteristics of these kind of tumours. They concluded that the majority of patients were male and the average age was about 64 years old. The antrum and pylorus are the common primary sites of tumour and there were AFP levels much higher than normal. Although there were no major symptoms sufficient to allow diagnosis of this type of cancer, epigastric pain and general fatigue, because of anaemia, were the most common symptoms. Most cases also had metastatic disease to the lymph nodes or liver. Data suggest than even early-stage hepatoid adenocarcinoma has an extremely poor prognosis.

Nowadays, the reasons for the poor prognosis are not clearly understood. One possibility is that hepatoid adenocarcinoma produces AAT (alpha-1 antitrypsin) and/or ACT (alpha-1 antichymotripsin) as well as AFP. AAT and ACT have immunosuppressive and protease-inhibitory properties that enhance invasiveness [16,17]. Also, AFP has a suppressive effect on lymphocyte transformation. Furthermore, these tumours are known to be resistant to chemotherapy [16,18].

The efficacy of palliative gastrectomy in patients with incurable advanced gastric cancer remains debatable. Previous reports suggested that palliative gastrectomy might be beneficial for younger patients (aged under 70 years) with one mestastatic site [19], while other clinicians argued that the survival advantage of palliative gastrectomy could be confirmed if the tumor spread was restricted to one site [20]. Another report showed that palliative gastrectomy combined with systemic chemotherapy improved survival in a group of patients with stage IV tumors [21]. While most of the studies published so far qualified the extent of the advanced gastric cancer, and found a survival benefit after resection if the number of metastatic sites of spread was limited to one or two [20-25], the literature is divided regarding the effect of resection on quality of life [26]. The presence of metastatic hepatoid adenocarcinoma of the stomach discovered at surgery may pose problems in surgical decision-making. There is a question as to whether liver involvement is secondary to the primary tumor of the stomach or the stomach involvement secondary to the primary tumor of the liver [27]. In patients in satisfactory general condition, with metastatic hepatoid adenocarcinoma of the stomach to the liver, whether diagnosed preoperatively or at surgery, we believe the stomach tumor should be resected if feasible for palliation.

Palliative chemotherapy should be advised to all patients with advanced gastric cancer and good performance status. Analysis of results of trials carried out until 2003 on this topic showed chemotherapy to be superior to best supportive care alone. Combination chemotherapy compared with monochemotherapy was also associated with significantly higher overall (complete plus partial) response rates but nevertheless resulted in similar survival. Cisplatin and 5-fluorouracil combination with or without epirrubicin used by then to represent the most effective regimens for advanced gastric cancer [28]. New combination regimens incorporating chemotherapeutic agents such as docetaxel, irinotecan, capecitabine and oxaliplatin have broadened perspectives for patients in terms of response rates, progression free and overall survival, and quality of life [29-32]. Capecitabine is an oral fluoropyrimidine already approved for colorrectal and breast cancer treatment which has been recently approved for the treatment of advanced gastric cancer in combination with a cisplatin containing regimen. The approval is based on the results of two randomised trials which demonstrated non-inferiority of capecitabine as compared to continuous infusion of fluorouracil in terms of overall survival, progression free survival, as well as of response rates [31,32]. In addition, by substituting flurorouracil for capecitabine, convenience of an oral treatment, and avoidance of possible complications and drawbacks related to continuous infusion are offered to the patients, with consequently gain in quality of life.

In conclusion, we report a case hepatoid adenocarcinoma of the stomach, a relatively rare entity, with well defined clinicopathological features, and usually associated to recognized poor prognosis, in which the realisation of palliative surgery and the administration of novel palliative systemic chemotherapy may have had major impact on survival.

## Consent

Wrtitten informed consent was obtained from the patient for publication of this case report and any accompanying images. A copy of the written informed consent is available for review by the Editor-in-Chief of this Journal.

## Competing interests

The authors declare that they have no competing interests.

## Authors' contributions

EGM designed and coordinated the case report. JGP conceived of the case report.VGO helped to draft the manuscript. FMP was responsable for surgery aspects. JARM was responsable for pathology assesment. MAM revised the manuscrpt critically for important intellectual content. All authors read and approved the final manuscript.
